# Prenatal paracetamol exposure is associated with shorter anogenital distance in male infants

**DOI:** 10.1093/humrep/dew196

**Published:** 2016-10-21

**Authors:** B.G. Fisher, A. Thankamony, I.A. Hughes, K.K. Ong, D.B. Dunger, C.L. Acerini

**Affiliations:** 1 Department of Paediatrics, University of Cambridge, Box 116, Level 8, Addenbrooke‘s Hospital, Hills Road, Cambridge CB2 0QQ, UK; 2 MRC Epidemiology Unit, University of Cambridge, Box 285, Institute of Metabolic Science, Cambridge Biomedical Campus, Cambridge CB2 0QQ, UK

**Keywords:** paracetamol, testicular dysgenesis syndrome, anogenital distance, testicular descent, endocrine disruption, toxicology

## Abstract

**STUDY QUESTION:**

What is the relationship between maternal paracetamol intake during the masculinisation programming window (MPW, 8–14 weeks of gestation) and male infant anogenital distance (AGD), a biomarker for androgen action during the MPW?

**SUMMARY ANSWER:**

Intrauterine paracetamol exposure during 8–14 weeks of gestation is associated with shorter AGD from birth to 24 months of age.

**WHAT IS ALREADY KNOWN:**

The increasing prevalence of male reproductive disorders may reflect environmental influences on foetal testicular development during the MPW. Animal and human xenograft studies have demonstrated that paracetamol reduces foetal testicular testosterone production, consistent with reported epidemiological associations between prenatal paracetamol exposure and cryptorchidism.

**STUDY DESIGN, SIZE, DURATION:**

Prospective cohort study (Cambridge Baby Growth Study), with recruitment of pregnant women at ~12 post-menstrual weeks of gestation from a single UK maternity unit between 2001 and 2009, and 24 months of infant follow-up. Of 2229 recruited women, 1640 continued with the infancy study after delivery, of whom 676 delivered male infants and completed a medicine consumption questionnaire.

**PARTICIPANTS/MATERIALS, SETTING, METHOD:**

Mothers self-reported medicine consumption during pregnancy by a questionnaire administered during the perinatal period. Infant AGD (measured from 2006 onwards), penile length and testicular descent were assessed at 0, 3, 12, 18 and 24 months of age, and age-specific Z scores were calculated. Associations between paracetamol intake during three gestational periods (<8 weeks, 8–14 weeks and >14 weeks) and these outcomes were tested by linear mixed models. Two hundred and twenty-five (33%) of six hundred and eighty-one male infants were exposed to paracetamol during pregnancy, of whom sixty-eight were reported to be exposed during 8–14 weeks. AGD measurements were available for 434 male infants.

**MAIN RESULTS AND THE ROLE OF CHANCE:**

Paracetamol exposure during 8–14 weeks of gestation, but not any other period, was associated with shorter AGD (by 0.27 SD, 95% CI 0.06–0.48, *P* = 0.014) from birth to 24 months of age. This reduction was independent of body size. Paracetamol exposure was not related to penile length or testicular descent.

**LIMITATIONS, REASONS FOR CAUTION:**

Confounding by other drugs or endocrine-disrupting chemicals cannot be discounted. The cohort was not fully representative of pregnant women in the UK, particularly in terms of maternal ethnicity and smoking prevalence. There is likely to have been misclassification of paracetamol exposure due to recall error.

**WIDER IMPLICATIONS OF THE FINDINGS:**

Our observational findings support experimental evidence that intrauterine paracetamol exposure during the MPW may adversely affect male reproductive development.

**STUDY FUNDING/COMPETING INTERESTS:**

This work was supported by a European Union Framework V programme, the World Cancer Research Fund International, the Medical Research Council (UK), the Newlife Foundation for Disabled Children, the Evelyn Trust, the Mothercare Group Foundation, Mead Johnson Nutrition, and the National Institute for Health Research Cambridge Comprehensive Biomedical Research Centre. The authors declare no conflict of interest.

## Introduction

Male genital disorders at birth (hypospadias and cryptorchidism) and reproductive disorders in adulthood (poor semen quality and testicular germ cell cancer) are common, and may be increasing in prevalence (Bay *et al*., 2006). Skakkebaek *et al*. (2001) proposed that these disorders could arise from foetal Leydig and Sertoli cell dysfunction, resulting in androgen insufficiency and impaired germ cell development—the testicular dysgenesis syndrome (TDS). There has been much interest in possible environmental causes of TDS, with recent attention turning to mild analgesics, including paracetamol (acetaminophen) (Kristensen *et al*., 2011). Paracetamol readily crosses the placenta (Rayburn *et al*., 1986) and is used during pregnancy by 30–70% of women in the Western world (Shaheen *et al*., 2002; Werler *et al*., 2005; Rebordosa *et al*., 2008; Kristensen *et al*., 2011; Snijder *et al*., 2012; Lupattelli *et al*., 2014; Vesterholm Lind *et al*., 2015). Leffers and colleagues were the first to demonstrate that gestational exposure of male rats to paracetamol inhibits masculinisation, through a reduction in testicular testosterone production (Kristensen *et al*., 2011, 2012). Subsequently, use of a xenograft model demonstrated that administration of paracetamol at the equivalent of a human therapeutic dose for 7 days reduces human fetal testicular testosterone production (van den Driesche *et al*., 2015), although another group had previously found no effect of 1–3 days of paracetamol exposure on testosterone production by human foetal testes *in vitro* (Mazaud-Guittot *et al*., 2013).

Epidemiological studies of gestational paracetamol exposure and TDS have focused on hypospadias and cryptorchidism, with no significant associations reported for the former (in adjusted models) and conflicting results for the latter (Aselton *et al*., 1985; Correy *et al*., 1991; Rebordosa *et al*., 2008; Feldkamp *et al*., 2010; Jensen *et al*., 2010; Kristensen *et al*., 2011; Wagner-Mahler *et al*., 2011; Snijder *et al*., 2012; Lind *et al*., 2013). Such studies require large cohorts to obtain sufficient numbers of these infrequent outcomes. Anogenital distance (AGD) is an alternative biomarker of foetal testicular function, which in animal models reflects androgen action during the masculinisation programming window (MPW), the developmental period during which sufficient androgen exposure must occur to ensure subsequent normal differentiation and growth of male reproductive organs (Welsh *et al*., 2008). In humans, the MPW is estimated to be 8–14 weeks of gestation (Welsh *et al*., 2008). A shorter AGD in males has been associated with cryptorchidism, hypospadias, less masculine play behaviour in childhood, and poor semen quality, infertility, small testes and low serum testosterone levels in adulthood (Dean and Sharpe, 2013; Thankamony *et al*., 2014; Pasterski *et al*., 2015).

Another measure that reflects androgen action during the MPW in rodents is penile length (Welsh *et al*., 2008), although in humans it has been associated only with hypospadias and cryptorchidism (Thankamony *et al*., 2009, 2014), not adult male reproductive outcomes (Eisenberg *et al*., 2011, 2012; Khan *et al*., 2012).

Our objective, therefore, was to investigate the relationship between maternal paracetamol intake during pregnancy, particularly during the postulated MPW, and male infant genital developmental outcomes, with the hypothesis that paracetamol exposure during the MPW, but not before or afterwards, reduce AGD, and may also shorten penile length and impair testicular descent. We also hypothesised that any paracetamol-related reduction in AGD in males would not be seen in females, who generate significantly lower levels of androgens *in utero* (Robinson *et al*., 1977). To our knowledge, no previous studies have examined these specific associations.

## Materials and Methods

### Study design

**Figure 1:**
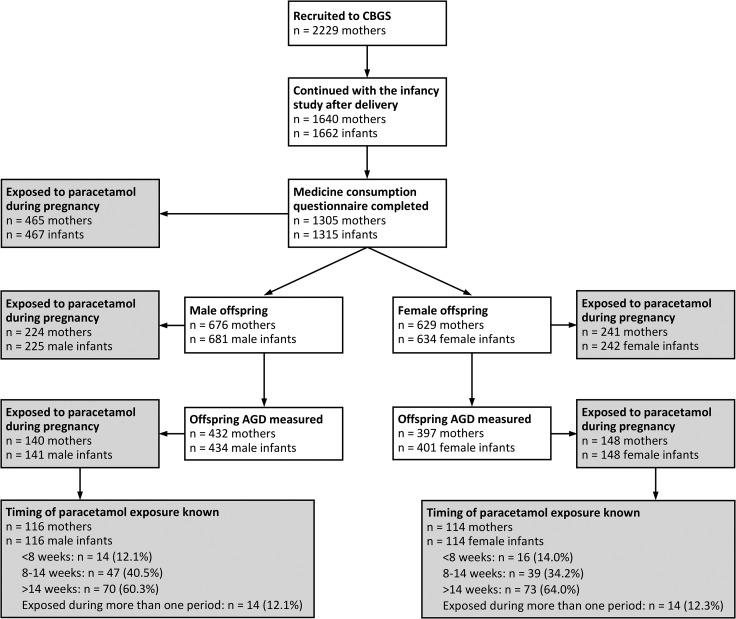
Flowchart for the study population. Abbreviations: CBGS, Cambridge Baby Growth Study; AGD, anogenital distance.

Mothers and infants were recruited to the Cambridge Baby Growth Study (CBGS), a large prospective cohort study (Prentice *et al*., 2015) (Fig. 1). Mothers (*n* = 2229) were recruited from routine ultrasound clinics at ~12 post-menstrual weeks of gestation at the Rosie Maternity Unit, Addenbrooke‘s Hospital, Cambridge, between April 2001 and March 2009. The unit supports a mixed urban and rural community. Mothers were excluded from the study if they were aged <16 years or were unable to give consent. Written informed consent was obtained from all mothers by a research nurse.

One thousand six hundred and forty women continued with the infancy study after delivery. For the analysis of male infant genital developmental outcomes (AGD, penile length and testicular descent), mother–infant dyads were included if the infant was male and the mother had completed a questionnaire during the perinatal period, including questions on drug exposure during pregnancy. This subset (676 mothers, 681 male offspring) was representative of the whole cohort with regard to maternal age, pre-pregnancy body mass index (BMI), deprivation index, ethnicity and parity, but mothers were less likely to be smokers (22/673 (3.3%) versus 64/953 (6.6%), corrected *χ*^2^ = 8.41, *P* = 0.004).

Measurement of AGD was introduced to the study protocol in March 2006, so only 434/681 (63.7%) male infants had AGD measured. This group was also representative of the entire CBGS cohort, apart from maternal smoking prevalence (10/430 (2.3%) versus 76/1130 (6.3%), corrected *χ*^2^ = 9.28, *P* = 0.002).

For the analysis of female infant AGD, mother–infant dyads were included if the infant was female, the mother had completed a medicine consumption questionnaire and AGD was measured. This subset (397 mothers, 401 female offspring) was similarly representative of the whole CBGS cohort, apart from maternal smoking prevalence (9/397 (2.3%) versus 77/1244 (6.2%), corrected *χ*^2^ = 8.55, *P* = 0.003).

### Maternal demographic characteristics and paracetamol exposure

A printed questionnaire was given to women at the time of recruitment with instructions that it should be completed in time for collection ‘after your baby is born’ (the time period was not further specified, but mothers were asked to record the date they completed the questionnaire). The questionnaire gathered information on maternal demographic characteristics, and also asked: ‘*Have you taken any medicine during this pregnancy?*’ Women who answered ‘yes’ were further instructed to complete a table with the following headings: ‘*Name*’, ‘*Disease*’, ‘*Daily Dose*’, ‘*No. of days*’ and ‘*Gestational Week(s)*’. The top row of the table displayed ‘*paracetamol*’ as an example medication. Women were considered to have been exposed to paracetamol if they reported taking ‘paracetamol’, ‘co-codamol’, ‘co-dydramol’ or ‘acetaminophen’. Paracetamol intake was categorised by gestational period, where specified (<8 weeks, 8–14 weeks or >14 weeks). We additionally collated data on gestational exposure to ibuprofen, aspirin, codeine and dihydrocodeine, but numbers of exposed male infants were too small for meaningful analysis (all ≤10).

### Examinations and anthropometric measurements

Infants were examined and measured as newborns, as far as possible in the first 2 weeks of life, either in hospital or at home visits. They were then re-examined during clinic visits at 3, 12, 18 and 24 months of age.

Birth weight as measured at delivery by midwives was taken from health records. Subsequent examinations and measurements were carried out by research nurses, as previously described (Thankamony *et al*., 2009). AGD, and for males, penile length and testicular descent distance, were measured to the nearest 0.1 mm using Vernier callipers (DialMax, Wiha Premium Tools, Schonach, Germany). AGD was measured from the centre of the anus to the junction of smooth perineal skin and rugated skin of the scrotum in males, and to the posterior convergence of the fourchette in females. Penile length was measured from the inferior edge of the pubic bone to the tip of the flaccid penis (excluding the foreskin). Testicular descent distance was measured from the superior edge of the pubic bone to the superior aspect of the testis after manipulation into the scrotum. Cryptorchidism was defined as one or more testes not present in the inferior half of the scrotum for at least a few moments after manipulation.

### Ethical approval

The study was approved by the local research ethics committee and adhered to the Declaration of Helsinki for Medical Research involving Human Subjects.

### Statistical analyses

Differences in maternal and infant characteristics were assessed using the *t*-test and Mann–Whitney *U* test for continuous variables, and chi-square test for categorical variables.

In view of the high correlation between left- and right-sided testicular descent distances at each time point (*r* = 0.89–0.93, *P* all < 0.0005), we present findings for the average of both distances.

AGD, penile length, testicular descent distance, body weight and gestation-corrected age at each time point (0, 3, 12, 18 and 24 months) approximated normal distributions. To effectively utilise repeated outcome measurements at 0–24 months, we employed *Z* scores, which represent the number of standard deviations (*σ*) an element (*X*) is from the population mean (*μ*) (*Z* score = (*X* − *μ*)/*σ*). We first generated *Z* scores, adjusted for gestation-corrected age, for AGD (males and females separately), penile length and testicular descent distance at each time point. We also calculated *Z* scores for body weight using British 1990 reference data (Freeman *et al*., 1995). We then used linear mixed models to test the relationships between paracetamol exposure and the repeated outcome measures. Separate models were generated for each gestational period (at any time in pregnancy, <8 weeks, 8–14 weeks and >14 weeks of gestation) and each outcome (AGD, penile length and testicular descent distance). Models using a first-order autoregressive covariance matrix to account for repeated and correlated measurements at different time points within an individual produced the best fit according to the Akaike Information Criterion (Akaike, 1974). We further adjusted for body weight *Z* score at each time point, and paracetamol exposure during other gestational periods, where relevant. We did not include other maternal covariates (age, deprivation index, BMI, fasting blood glucose, parity, smoking, method of delivery or ethnicity) in the models, as they were not significantly associated with AGD, penile length or testicular descent distance.

Differences in prevalence of cryptorchidism by paracetamol exposure were assessed using chi-square test followed by logistic regression with adjustment for body weight and gestation-corrected age.

We performed a sensitivity analysis in the majority of male infants with AGD measurements who were born full-term (gestational age ≥37 weeks) and not low birth weight (birth weight ≥2.5 kg) (*n* = 414).

All *P*-values were two-sided and *P* < 0.05 was considered statistically significant. Data were analysed using SPSS, version 21.0 (IBM Corporation, New York, USA).

## Results

Six hundred and eighty-one male offspring of six hundred and seventy-six mothers were included in the analysis of male infant genital developmental outcomes (Fig. 1). The mean ± SD post-menstrual age at completion of the questionnaire, where known (*n* = 611 mothers), was 39.8 ± 10.8 weeks. A total of 313/611 (52.1%) questionnaires were completed before birth and 519/611 (84.9%) before 3 months postnatally.

Two hundred and twenty-five male infants (33.0%) were reported as being exposed to paracetamol at least once during pregnancy. Of the 224 mothers who took paracetamol, data on timings of exposure were available for 186: 25 (13.4%) took paracetamol at <8 weeks of gestation, 68 (36.6%) during 8–14 weeks and 117 (62.9%) at >14 weeks; 23 mothers (12.4%) took paracetamol during more than one of the three periods. The median total dose consumed during pregnancy was 3.0 g (range 0.5–360.0 g). The most common indications for taking paracetamol were headache or migraine (133/224, 59.4%) and infection (41/224, 18.3%) (Supplementary Table I).

There were no differences in any demographic or birth variables between mothers or male infants by exposure to paracetamol at any time during pregnancy (Table I), or by exposure at <8 weeks, 8–14 weeks or >14 weeks of gestation (data not shown). Only one infant had hypospadias.

**Table I:** Characteristics of male infants included in the analysis of male infant genital developmental outcomes (*n* = 681) and their mothers (*n* = 676). Values are mean ± SD or median (interquartile range) for continuous variables, *n* (%) for categorical variables.

Characteristics	Exposed to paracetamol at any time during pregnancy	Not exposed to paracetamol at any time during pregnancy	*P*^a^
**Mothers**	***n* = 224**	***n* = 452**	
Age (years)	33.5 ± 3.9	33.5 ± 3.9	0.82
Pre-pregnancy BMI (kg/m^2^)	23.5 (21.5, 26.0)	22.8 (20.9, 26.0)	0.08
Ethnicity^b^			
White	129 (95.6)	297 (96.1)	0.99
Other	6 (4.4)	12 (3.9)	
Current smoker			
No	216 (96.4)	437 (96.9)	0.93
Yes	8 (3.6)	14 (3.1)	
Parity			
0	95 (42.4)	201 (44.5)	0.81
1	92 (41.1)	184 (40.7)	
≥2	37 (16.5)	67 (14.8)	
Index of multiple deprivation (units)	7.7 (6.4, 10.5)	7.6 (6.4, 11.6)	0.98
Completion of questionnaire (post-menstrual weeks)	39.7 ± 10.8	40.2 ± 10.9	0.54
**Male infants**	***n* = 225**	***n* = 456**	
Gestation (weeks)	39.8 ± 1.3	39.8 ± 1.7	0.75
Birth weight (kg)	3.56 ± 0.48	3.58 ± 0.55	0.58
Birth AGD (cm)^c^	1.96 ± 0.61	1.96 ± 0.59	0.99
Birth penile length (cm)	3.14 ± 0.54	3.14 ± 0.49	0.99
Birth testicular descent distance (cm)			
Right	5.07 ± 0.68	5.12 ± 0.67	0.45
Left	5.08 ± 0.73	5.13 ± 0.70	0.51
Congenital cryptorchidism^d^			
No	210 (95.0)	407 (94.9)	0.99
Yes	11 (5.0)	22 (5.1)	

Abbreviation: AGD, anogenital distance.

^a^*P-*values: comparing exposed and unexposed mothers/infants. Mann–Whitney *U* test for pre-pregnancy BMI and index of multiple deprivation; *t*-test for other continuous variables; chi-square test for categorical variables (with Yates’ continuity correction for 2 × 2 tables).

^b^Data on ethnicity are missing for 232 mothers.

^c^Data on birth AGD are missing for 389 infants.

^d^Data on congenital cryptorchidism are missing for 31 infants.

Overall in CBGS, 465 of 1305 mothers (35.6%) consumed paracetamol during pregnancy, exposing 467 of 1315 male and female infants (35.5%). The difference between the proportions of male and female infants exposed to paracetamol *in utero* (225/681 (33.0%) versus 242/634 (38.2%)) narrowly failed to achieve statistical significance (corrected *χ*^2^ = 3.55, *P* = 0.06). After excluding those for whom timings of exposure were unavailable (*n* = 93 mothers), 125 of 1212 mothers (10.3%) reported taking paracetamol in the first trimester (12 weeks) of pregnancy.

### Associations between paracetamol exposure and AGD in males

**Figure 2:**
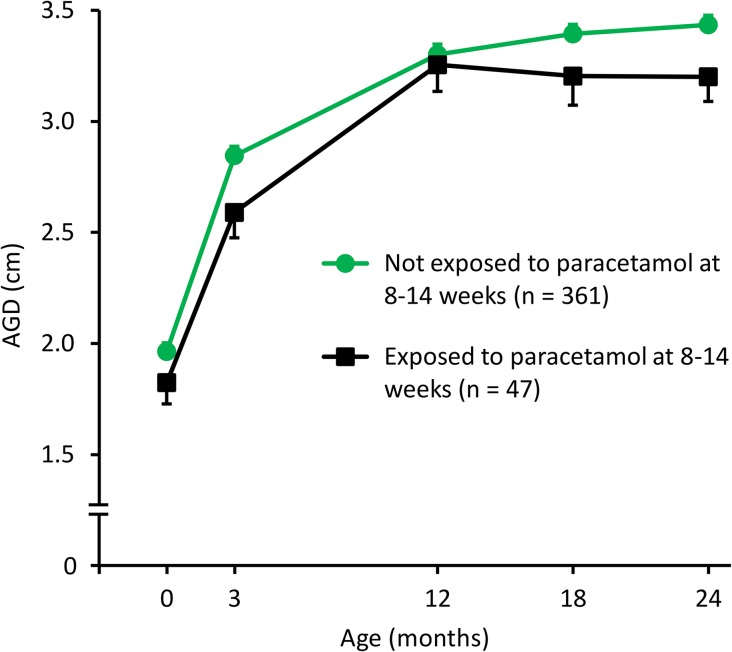
Anogenital distance (AGD) at 0–24 months in relation to paracetamol exposure during 8–14 weeks of gestation in male infants. A break has been included in the y axis to more clearly demonstrate the differences between the groups. Data represent the means and error bars represent SEM. The total number of infants (*n* = 408) is less than the number of male infants with AGD and paracetamol exposure data in Cambridge Baby Growth Study (CBGS) (*n* = 434) because it does not include infants who were exposed to paracetamol during pregnancy but at an unknown time point.

Four hundred and thirty-four male infants had AGD measured at one or more time points (mean 3.8 AGD measurements per infant), of whom 141 (32.5%) were exposed to paracetamol at least once during pregnancy (Fig. 1). Of these, 47 were reported to be exposed during 8–14 weeks of gestation, of whom 1 infant was also exposed at both <8 weeks and >14 weeks and another 11 infants were exposed at >14 weeks. In the unadjusted model, paracetamol exposure during 8–14 weeks of gestation was associated with an AGD between birth and 24 months of age that was shorter by 0.28 SD (95% CI 0.06–0.49, *P* = 0.012) (Supplementary Table II). This difference was not explained by body weight or additional exposure to paracetamol at <8 or >14 weeks (adjusted model: *P* = 0.014) (Table II). Further addition of a body weight *Z* score × exposure interaction term also did not attenuate the association (data not shown). The reduction in AGD in male infants exposed to paracetamol during 8–14 weeks appeared to be consistent from birth to 24 months of age (Fig. 2, Supplementary Fig. 1, Supplementary Table III), and there was no significant interaction between paracetamol exposure during 8–14 weeks and time point of AGD measurement when we added this interaction term to the model (*P* = 0.98). Exposure during any other gestational period was not associated with AGD (Table II).

**Table II:** Adjusted linear mixed models exploring the associations between gestational exposure to paracetamol and male genital developmental outcomes at all time points (0–24 months) (*n* = 434 for AGD, *n* = 677 for penile length, *n* = 662 for testicular descent distance).

Measure	Exposure to paracetamol at any time			Exposure to paracetamol at <8 weeks			Exposure to paracetamol during 8–14 weeks			Exposure to paracetamol at >14 weeks		
	*n* (exposed, not exposed)	Parameter estimate^a^	*P*^b^	*n* (exposed, not exposed)	Parameter estimate^a^	*P*^b^	*n* (exposed, not exposed)	Parameter estimate^a^	*P*^b^	*n* (exposed, not exposed)	Parameter estimate^a^	*P*^b^
AGD^c,d^	141, 293	−0.014 (−0.156, 0.127)	0.84	14, 394	−0.065 (−0.423, 0.295)	0.72	47, 361	−0.265 (−0.476, −0.055)	0.014*	69, 339	0.084 (−0.090, 0.258)	0.34
Penile length^c,e^	225, 452	−0.051 (−0.172, 0.069)	0.40	25, 612	−0.255 (−0.559, 0.049)	0.10	68, 569	−0.119 (−0.310, 0.072)	0.22	116, 521	0.063 (−0.091, 0.218)	0.42
Testicular descent distance^c,f^	222, 440	−0.086 (−0.193, 0.021)	0.12	25, 600	−0.161 (−0.427, 0.105)	0.24	68, 557	0.004 (−0.163, 0.170)	0.97	116, 509	−0.070 (−0.204, 0.065)	0.31

Abbreviations: AGD, anogenital distance; CI, confidence interval. *P < 0.05 for exposed versus not exposed.

^a^Parameter estimate (95% CI).

^b^*P-*values: comparing exposed and unexposed infants for the relevant gestational periods.

^c^Adjusted for time point and body weight *Z* score at time of measurement. Additionally, adjusted for exposure during other gestational periods where applicable; e.g. models for exposure to paracetamol during 8–14 weeks: adjusted for exposure at <8 weeks and >14 weeks.

^d^Sex-specific AGD *Z* scores adjusted for gestation-corrected age at time of measurement.

^e^Penile length *Z* scores adjusted for gestation-corrected age at time of measurement.

^f^Testicular descent distance *Z* scores adjusted for gestation-corrected age at time of measurement.

In a sensitivity analysis restricted to the 414 male infants with AGD measurements who were born full-term and not low birth weight, exposure to paracetamol during 8–14 weeks of gestation was also associated with shorter AGD, by 0.28 SD (95% CI 0.07–0.49, *P* = 0.011) in the model adjusted for body weight and exposure at <8 and >14 weeks.

### Associations between paracetamol exposure and other male genital outcomes

Data on penile length were available for 677 infants (mean 4.0 measurements per infant) and data on testicular descent distance were available for 662 infants (mean 3.6 measurements per infant). Neither penile length nor testicular descent distance was significantly associated with paracetamol exposure during any gestational period (Table II, Supplementary Table II, Supplementary Table IV). We also found no significant association between paracetamol exposure and cryptorchidism at any postnatal time point (data not shown), although our numbers were small (overall prevalence 33/650 (5.1%) at birth and 62/424 (14.6%) at any age).

### Associations between paracetamol exposure and AGD in females

Data on AGD were available for 401 female infants (mean 3.8 measurements per infant), of whom 148 (36.9%) were reported as being exposed to paracetamol at least once during pregnancy (Fig. 1, Supplementary Table V). Gestational paracetamol exposure (at any time, <8 weeks, 8–14 weeks or >14 weeks) was not associated with AGD between birth and 24 months of age (data not shown). Female infants exposed to paracetamol did, however, have a significantly longer gestation than unexposed female infants (40.1 ± 1.2 weeks versus 39.7 ± 1.6 weeks, *P* = 0.004) (Supplementary Table V), with a smaller proportion of exposed infants being born prematurely (i.e. at <37 weeks, 1/148 (0.7%) versus 12/253 (4.7%), corrected *χ*^2^ = 3.71, *P* = 0.054).

## Discussion

### Summary of main findings

In this prospective study, we found a significant negative association between reported paracetamol exposure during the postulated MPW (8–14 weeks of gestation) and AGD, a marker of intrauterine androgen action (Welsh *et al*., 2008), in male infants. This association was consistent postnatally across 0–24 months of age, and was independent of body weight.

### Interpretation of findings

Although the association between paracetamol exposure and AGD was modest in size and not highly significant, it may represent a true effect of paracetamol on foetal androgen action, for several reasons. Firstly, the association was robust to a more stringent sensitivity analysis (full-term males who were not low birth weight). Secondly, the relationship was found only for paracetamol exposure during 8–14 weeks of gestation, and persisted after adjustment for exposure at <8 and >14 weeks—consistent with extrapolations from animal studies that this period represents the programming window for normal reproductive tract masculinisation (Welsh *et al*., 2008). Thirdly, our findings are consistent with experimental models of paracetamol exposure: administration to rats of paracetamol at 150 mg/kg/day during the MPW resulted in a reduction in AGD of ~2 SD (Kristensen *et al*., 2011); and a therapeutic regimen of paracetamol (20 mg/kg, three times daily) for 7 days was sufficient to decrease human foetal testicular testosterone production by 45% in a xenograft model (van den Driesche *et al*., 2015). Fourthly, a trend towards reduced AGD at 3 months of age in Danish male infants exposed to paracetamol during the first and second trimesters has recently been reported in abstract form (Vesterholm Lind *et al*., 2015). Fifthly, three epidemiological studies have associated gestational paracetamol exposure with another indicator of foetal androgen action, cryptorchidism (Jensen *et al*., 2010; Kristensen *et al*., 2011; Snijder *et al*., 2012); indeed, one study reported a strongest association with prolonged exposure during 8–14 weeks of gestation (Jensen *et al*., 2010). Sixthly, we found no effect of paracetamol exposure on AGD in girls, consistent with its actions being mediated via decreased testicular testosterone production rather than a direct effect on the perineum or overall growth, and making statistical confounding a less likely explanation for the association seen in boys. Finally, we observed a concomitant but non-significant shortening of penile length with paracetamol exposure during 8–14 weeks, providing further evidence of possible impaired androgenisation.

Arguing against an anti-androgenic effect of paracetamol is a lack of significant association with testicular descent distance or cryptorchidism in this study, compared with previous conflicting reports. One possible explanation is that our study was underpowered to detect an effect of paracetamol on testicular descent: the only previous epidemiological studies to demonstrate an association with cryptorchidism recruited 2800–47 400 boys (Jensen *et al*., 2010; Kristensen *et al*., 2011; Snijder *et al*., 2012), considerably more than our 681. Furthermore, testicular descent is influenced by other hormones than androgens (Hughes and Acerini, 2008), so not all cases of cryptorchidism will be due to foetal androgen deprivation, and the strength of any relationship with paracetamol exposure may therefore be weaker than that of AGD (Dean and Sharpe, 2013).

The mechanism by which paracetamol may disrupt foetal androgenisation remains unclear. Paracetamol is a cyclooxygenase (COX)-2 inhibitor (Kristensen *et al*., 2012), and early work with other COX inhibitors implicated the arachidonic acid cascade in the masculinisation of male mouse embryos (Gupta and Goldman, 1986). However, parallel determinations of *ex vivo* testicular testosterone and prostaglandin production have indicated that the anti-androgenic effects of paracetamol are independent of prostaglandins, and furthermore are not related to insulin-like 3 (INSL3) production, Leydig cell numbers or gonocyte apoptosis (Kristensen *et al*., 2012). Recent *in vivo* work has demonstrated downregulation of the steroidogenic enzymes Cyp11a1 and Cyp17a1 in the testes of rat fetuses exposed to high-dose paracetamol (350 mg/kg/day) during the MPW (van den Driesche *et al*., 2015), which is supported by steroidogenic profiling data showing impaired conversion of progesterone to 17α-hydroxyprogesterone by CYP17A1, and possibly other downstream enzymes, in human adrenocortical carcinoma cells exposed to paracetamol *in vitro* (Holm *et al*., 2015). The mechanisms by which paracetamol might alter gene expression, however, are unknown.

An anti-androgenic effect of paracetamol during the MPW could have important public health implications, for several reasons. Firstly, paracetamol is used during the first 20 weeks of gestation by 54% of women in the UK (Shaheen *et al*., 2002), resulting in the exposure of large numbers of male infants. Secondly, there is limited evidence from epidemiological studies that deficiency in foetal androgens may be associated with hypospadias and cryptorchidism at birth, and infertility, poor semen quality, reduced serum testosterone levels and prostate cancer in adulthood (Dean and Sharpe, 2013; Thankamony *et al*., 2014). Thus, paracetamol exposure could potentially be contributing to the apparent increase in prevalence of these disorders (Bay *et al*., 2006). Finally, paracetamol would not be acting alone on foetal androgenisation: pregnant women are ubiquitously exposed to numerous other anti-androgenic compounds, which may exert stronger effects on male reproductive development in combination than individually (Christiansen *et al*., 2012).

We incidentally found that gestational paracetamol exposure in females, but not males, was associated with a significantly longer gestation (by an average of 2.8 days) and a trend towards a lower incidence of preterm birth (0.7% versus 4.7%). Remarkably, a retrospective Hungarian study reported an identical increase in mean gestational age of infants exposed to paracetamol during pregnancy (2.8 days), with a significant reduction in preterm deliveries (3.5% versus 9.2%), although they did not distinguish between male and female offspring (Czeizel *et al*., 2005). The authors speculated that paracetamol may prolong pregnancy via a reduction in prostacyclin synthesis, consistent with findings from a randomised controlled trial showing that low-dose aspirin (another COX inhibitor) also reduced preterm delivery (CLASP (Collaborative Low-dose Aspirin Study in Pregnancy) Collaborative Group, 1994). However, another observational study found no significant association between paracetamol prescriptions and preterm birth (Thulstrup *et al*., 1999), and a large prospective study reported that women using paracetamol during the third trimester had a significantly *increased* risk of preterm delivery (although in subgroup analyses this persisted only for mothers with pre-eclampsia, who may have been taking paracetamol for hypertension-related headache) (Rebordosa *et al*., 2009). Given the above, the fact that the association was not seen in males in our study, the repeated statistical testing we have undertaken without corrections, and the likely influence of length bias (infants born at term had a higher probability of paracetamol exposure because of the longer duration of the third trimester), we feel on balance that our result likely reflects chance rather than a true effect of paracetamol on gestational length.

We also incidentally found a non-significant trend towards a higher prevalence of intrauterine paracetamol exposure in female infants (38%) than in male infants (33%). We can see no way in which such exposure would alter the sex ratio, which is determined at conception, but other explanations (such as differential indications for analgesic consumption in mothers carrying male and female offspring) cannot be excluded completely.

### Limitations and strengths of the study

Our study has several limitations. Firstly, although we believe that confounding by indication for paracetamol consumption is unlikely, we cannot discount confounding by other drugs or endocrine-disrupting chemicals. Secondly, our sample size was relatively small (*n* = 434 for male AGD measurements, with missing data points), which limited the study‘s precision and statistical power. It also precluded analyses of dose–response relationships for paracetamol and AGD, the effects of other mild analgesics, or the association between paracetamol exposure and hypospadias, and may have resulted in type-II errors in our analyses of other male genital developmental outcomes. Thirdly, our cohort was not fully representative of pregnant women in the UK, particularly in terms of maternal ethnicity and smoking prevalence, and mothers who completed the questionnaire differed significantly in smoking prevalence from those who did not, raising the possibility of selection bias and limiting the generalisability of our results. Finally, there is likely to have been misclassification of paracetamol exposure due to recall error. We collected data on medication consumption from a single questionnaire given to mothers at recruitment (~12 weeks of gestation) but completed an average of 6 months after the MPW, by which time mothers may have been unable to accurately recall their earlier paracetamol intake. Certainly, the proportion of mothers (of all infants) in CBGS who admitted to gestational paracetamol consumption (36%) was lower than has been reported in most studies that administered questionnaires during, rather than after, pregnancy (40–54%) (Shaheen *et al*., 2002; Rebordosa *et al*., 2008; Kristensen *et al*., 2011; Vesterholm Lind *et al*., 2015), with a Netherlands study being the one exception (30%) (Snijder *et al*., 2012). Furthermore, MacLeod and colleagues reported that only 31% of women correctly remembered first-trimester exposure to over-the-counter medications 15 months later (Feldman *et al*., 1989). However, arguing against time-dependent underreporting is the fact that the proportion of mothers in our study who recalled taking paracetamol in the first 12 weeks of pregnancy (10%) was similar to those from studies that administered questionnaires during the first trimester (9–30%) (Rebordosa *et al*., 2008; Snijder *et al*., 2012). In addition, studies that administered questionnaires *postnatally* have tended to describe higher, rather than lower, prevalences of gestational paracetamol consumption (49–70%), including during the first trimester (47–54%) (Werler *et al*., 2005; Feldkamp *et al*., 2010; Lupattelli *et al*., 2014)—although this may in part be explained by the different populations being studied (generally higher prevalence in North America versus lower prevalence in Europe). In support of our study design, we included paracetamol as an example medication in our questionnaire, which may increase reporting of mild analgesia consumption by >85% (Kristensen *et al*., 2011). Additionally, underreporting of paracetamol consumption would be expected to bias results towards the null hypothesis, which argues against the association with AGD being a type-I error.

Our study's strengths include the following: use of a well-characterised birth cohort; ascertainment of gestational paracetamol intake before AGD measurement in the majority of cases; use of AGD as our primary measure of intrauterine androgen action, which is more sensitive than hypospadias or cryptorchidism (Kristensen *et al*., 2011); and measurement of AGD from the centre of anus to the posterior edge of the scrotum (rather than to the anterior or posterior base of the penis), which has been shown to be the most reliable and repeatable measurement of AGD (Dean and Sharpe, 2013).

### Conclusion and implications

Our study provides novel and interesting data that support the hypothesis that intrauterine paracetamol exposure during the MPW may adversely affect male reproductive development. However, these findings are preliminary, and will need to be confirmed by larger, carefully-designed prospective studies using paracetamol exposure data collected concurrently throughout pregnancy.

## Supplementary data

Supplementary data are available at http://humrep.oxfordjournals.org/.

Supplementary Data
