# Supplementary material for: Prenatal paracetamol exposure is associated with shorter anogenital distance in male infants
Source: Hum Reprod. 2016 Oct 21;31(11):2642–50. doi: 10.1093/humrep/dew196 (PMC5088633; doi:10.1093/humrep/dew196)
Supplement: Supplementary Data [file supp_31_11_2642__index.html]

Prenatal paracetamol exposure is associated with shorter anogenital distance in male infants — Supplementary Data 

# Prenatal paracetamol exposure is associated with shorter anogenital distance in male infants

## Supplementary Data

Supplementary Data

- Supplementary Data - pdf file
- Supplementary Data - pdf file
- Supplementary Data - pdf file
- Supplementary Data - pdf file
- Supplementary Data - pdf file
- Supplementary Data - pdf file
