# Supplementary material for: Prenatal paracetamol exposure is associated with shorter anogenital distance in male infants
Source: Hum Reprod. 2016 Oct 21;31(11):2642–50. doi: 10.1093/humrep/dew196 (PMC5088633; doi:10.1093/humrep/dew196)
Supplement: Supplementary Data [file supp_dew196_dew196_suppl_figure1.pdf]

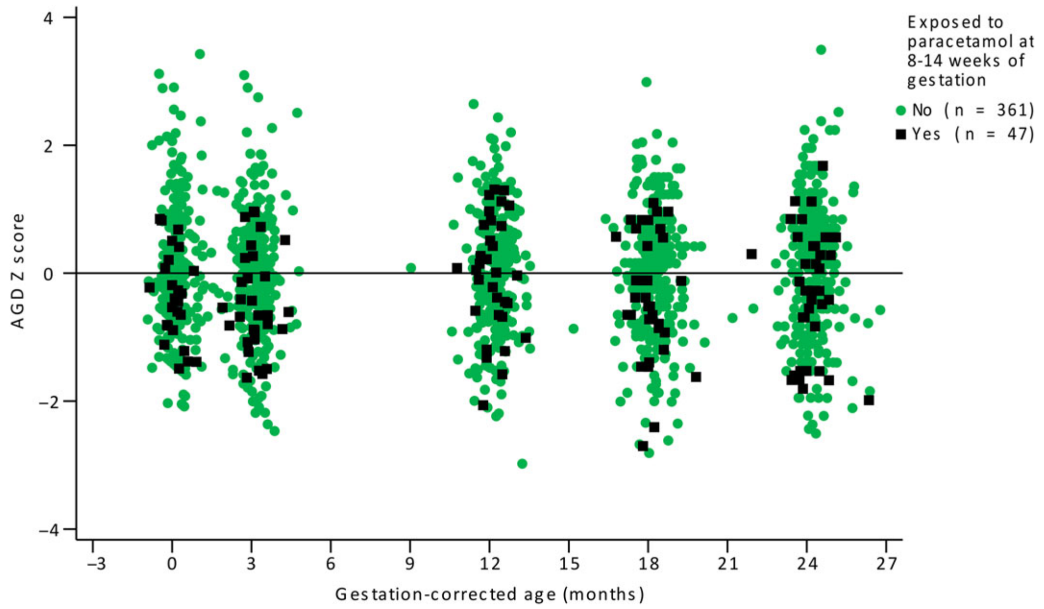

Supplementary Figure 1. Male infant anogenital distance (AGD) *Z* scores by gestation-corrected age at examination, labelled according to exposure to paracetamol during 8–14 weeks of gestation. The total number of infants ($n = 408$) is less than the number of male infants with AGD and paracetamol exposure data in CBGS ($n = 434$) because it does not include infants who were exposed to paracetamol during pregnancy but at an unknown time point.
