# Supplementary material for: Prenatal paracetamol exposure is associated with shorter anogenital distance in male infants
Source: Hum Reprod. 2016 Oct 21;31(11):2642–50. doi: 10.1093/humrep/dew196 (PMC5088633; doi:10.1093/humrep/dew196)
Supplement: Supplementary Data [file supp_dew196_dew196_suppl_table1.pdf]

Human Reproduction, 2016
doi:10.1093/humrep/dew196

human reproduction

SUPPLEMENTARY DATA

Supplementary Table SI **Reasons for taking paracetamol in pregnancy (224 mothers of 225 male infants).**

Reason	Number of women (%)
Headache or migraine	133 (59.4)
Infection, e.g. cold, flu, ear infection, pharyngitis, sinusitis, toothache, urinary tract infection, fever	41 (18.3)
Not stated or non-specific, e.g. pain relief, general pains	31 (13.8)
Musculoskeletal pain, e.g. back pain, muscle pain, sciatica, pelvic girdle pain, whiplash	13 (5.8)
Other, e.g. early labour pains, fibroids, postoperative pain, stomach cramps, varicose veins	6 (2.7)
