# Supplementary material for: Prenatal paracetamol exposure is associated with shorter anogenital distance in male infants
Source: Hum Reprod. 2016 Oct 21;31(11):2642–50. doi: 10.1093/humrep/dew196 (PMC5088633; doi:10.1093/humrep/dew196)
Supplement: Supplementary Data [file supp_dew196_dew196_suppl_table2.pdf]

**Supplementary Table SII** **Crude linear mixed models exploring the associations between gestational exposure to paracetamol and male genital developmental outcomes at all time points (0–24 months) (*n* = 434 for AGD, *n* = 677 for penile length, *n* = 662 for testicular descent distance).**

| Measure | Exposure to paracetamol at any time | | | Exposure to paracetamol at <8 weeks | | | Exposure to paracetamol during 8–14 weeks | | | Exposure to paracetamol at >14 weeks | | |
|---|---|---|---|---|---|---|---|---|---|---|---|---|
| | *n* (exposed, not exposed) | Parameter estimate[a] | *P*[b] | *n* (exposed, not exposed) | Parameter estimate[a] | *P*[b] | *n* (exposed, not exposed) | Parameter estimate[a] | *P*[b] | *n* (exposed, not exposed) | Parameter estimate[a] | *P*[b] |
| AGD[c,d] | 141,293 | −0.022 (−0.166, 0.121) | 0.76 | 14, 394 | −0.044 (−0.414, 0.327) | 0.82 | 47, 361 | −0.275 (−0.488, −0.062) | **0.012**[*] | 70, 339 | 0.058 (−0.119, 0.235) | 0.52 |
| Penile length[c,e] | 225,452 | −0.052 (−0.173, 0.068) | 0.39 | 25, 612 | −0.258 (−0.562, 0.046) | 0.10 | 68, 569 | −0.091 (−0.280, 0.098) | 0.34 | 117, 521 | 0.058 (−0.094, 0.211) | 0.45 |
| Testicular descent distance[c,f] | 222,440 | −0.084 (−0.195, 0.027) | 0.14 | 25, 600 | −0.149 (−0.426, 0.128) | 0.29 | 68, 557 | −0.009 (−0.180, 0.162) | 0.92 | 117, 509 | −0.061 (−0.200, 0.077) | 0.38 |

Abbreviations: AGD, anogenital distance.

[a]Parameter estimate (95% CI).

[b]*P* values: comparing exposed and unexposed infants for the relevant gestational periods.

[c]Adjusted for time point of measurement.

[d]Sex-specific AGD *Z* scores adjusted for gestation-corrected age at time of measurement.

[e]Penile length *Z* scores adjusted for gestation-corrected age at time of measurement.

[f]Testicular descent distance *Z* scores adjusted for gestation-corrected age at time of measurement.

[*]$P < 0.05$ for exposed versus not exposed.
