# Supplementary material for: Prenatal paracetamol exposure is associated with shorter anogenital distance in male infants
Source: Hum Reprod. 2016 Oct 21;31(11):2642–50. doi: 10.1093/humrep/dew196 (PMC5088633; doi:10.1093/humrep/dew196)
Supplement: Supplementary Data [file supp_dew196_dew196_suppl_table3.pdf]

Supplementary Table SIII AGD (cm) at 0–24 months by gestational exposure to paracetamol in male infants (n = 434, with missing data points).

Month	Exposure to paracetamol at any time			Exposure to paracetamol at <8 weeks			Exposure to paracetamol during 8–14 weeks			Exposure to paracetamol at >14 weeks		
	Yes	No	P^a	Yes	No	P^a	Yes	No	P^a	Yes	No	P^a
0	1.96 ± 0.61 (91)	1.96 ± 0.59 (201)	0.84	1.93 ± 0.58 (10)	1.95 ± 0.60 (267)	0.88	1.82 ± 0.51 (28)	1.96 ± 0.61 (249)	0.28	2.01 ± 0.68 (48)	1.93 ± 0.58 (229)	0.39
3	2.82 ± 0.82 (100)	2.84 ± 0.68 (216)	0.92	2.64 ± 0.75 (11)	2.83 ± 0.68 (286)	0.23	2.60 ± 0.65 (29)	2.85 ± 0.68 (268)	0.09	2.89 ± 0.67 (52)	2.81 ± 0.69 (246)	0.34
12	3.38 ± 0.75 (107)	3.28 ± 0.81 (234)	0.24	3.43 ± 0.52 (10)	3.29 ± 0.78 (308)	0.53	3.25 ± 0.69 (33)	3.30 ± 0.79 (285)	0.82	3.40 ± 0.67 (55)	3.28 ± 0.80 (264)	0.25
18	3.32 ± 0.83 (107)	3.40 ± 0.71 (231)	0.29	3.22 ± 0.42 (10)	3.38 ± 0.74 (304)	0.50	3.20 ± 0.78 (35)	3.39 ± 0.72 (279)	0.15	3.32 ± 0.86 (53)	3.38 ± 0.70 (262)	0.58
24	3.34 ± 0.66 (108)	3.42 ± 0.76 (238)	0.41	3.46 ± 0.39 (11)	3.41 ± 0.74 (314)	0.72	3.21 ± 0.69 (37)	3.43 ± 0.73 (288)	0.09	3.42 ± 0.65 (52)	3.41 ± 0.75 (274)	0.79

Values are mean ± SD (n).

[a]One-way ANCOVA, with body weight and gestation-corrected age at the time of measurement as covariates.
