# Supplementary material for: Prenatal paracetamol exposure is associated with shorter anogenital distance in male infants
Source: Hum Reprod. 2016 Oct 21;31(11):2642–50. doi: 10.1093/humrep/dew196 (PMC5088633; doi:10.1093/humrep/dew196)
Supplement: Supplementary Data [file supp_dew196_dew196_suppl_table4.pdf]

Supplementary Table SIV **Penile length (cm) at 0–24 months by gestational exposure to paracetamol (n = 677, with missing data points).**

Month	Exposure to paracetamol at any time			Exposure to paracetamol at <8 weeks			Exposure to paracetamol during 8–14 weeks			Exposure to paracetamol at >14 weeks		
	**Yes**	**No**	P^a	**Yes**	**No**	P^a	**Yes**	**No**	P^a	**Yes**	**No**	P^a
0	3.14 ± 0.54 (216)	3.14 ± 0.49 (432)	0.95	2.99 ± 0.53 (24)	3.14 ± 0.51 (589)	0.17	3.12 ± 0.50 (67)	3.14 ± 0.51 (546)	0.77	3.18 ± 0.57 (114)	3.12 ± 0.49 (500)	0.30
3	3.50 ± 0.54 (205)	3.51 ± 0.51 (422)	0.89	3.38 ± 0.80 (22)	3.52 ± 0.51 (568)	0.45	3.47 ± 0.49 (64)	3.52 ± 0.52 (526)	0.49	3.57 ± 0.50 (106)	3.50 ± 0.52 (485)	0.23
12	3.75 ± 0.52 (178)	3.75 ± 0.58 (375)	0.92	3.55 ± 0.57 (17)	3.76 ± 0.56 (505)	0.15	3.67 ± 0.57 (59)	3.76 ± 0.56 (463)	0.28	3.78 ± 0.49 (94)	3.74 ± 0.58 (429)	0.51
18	3.90 ± 0.62 (156)	4.04 ± 0.64 (316)	**0.017***	3.73 ± 0.58 (19)	4.02 ± 0.64 (424)	0.06	3.85 ± 0.70 (50)	4.02 ± 0.63 (393)	0.08	4.01 ± 0.59 (77)	4.01 ± 0.65 (367)	0.98
24	4.14 ± 0.65 (129)	4.15 ± 0.60 (288)	0.81	4.14 ± 0.63 (16)	4.16 ± 0.62 (374)	0.89	4.10 ± 0.68 (42)	4.17 ± 0.61 (348)	0.50	4.21 ± 0.66 (58)	4.15 ± 0.61 (333)	0.54

Values are mean ± SD (n).

[a]Penile length at 0 months: one-way ANCOVA, with gestation-corrected age at the time of measurement as covariate. Penile length at 3, 12, 18, and 24 months: independent samples t-test.

*$P < 0.05$ for exposed versus not exposed.
