# Supplementary material for: Prenatal paracetamol exposure is associated with shorter anogenital distance in male infants
Source: Hum Reprod. 2016 Oct 21;31(11):2642–50. doi: 10.1093/humrep/dew196 (PMC5088633; doi:10.1093/humrep/dew196)
Supplement: Supplementary Data [file supp_dew196_dew196_suppl_table5.pdf]

Supplementary Table SV **Characteristics of female infants included in the analysis of female AGD ($n = 401$) and their mothers ($n = 397$).**

Characteristics	Exposed to paracetamol at any time during pregnancy	Not exposed to paracetamol at any time during pregnancy	P[a]
**Mothers**	***n* = 148**	***n* = 249**	
Age (years)	33.8 ± 4.4	33.7 ± 4.3	0.85
Pre-pregnancy BMI (kg/m^2)	23.7 (21.0, 26.0)	22.6 (20.9, 25.4)	0.16
Ethnicity[b]			
White	133 (97.8)	207 (93.2)	0.10
Other	3 (2.2)	15 (6.8)	
Current smoker			
No	144 (97.3)	244 (98.0)	0.92
Yes	4 (2.7)	5 (2.0)	
Parity			
0	57 (38.5)	99 (39.8)	0.75
1	59 (39.9)	104 (41.8)	
≥2	32 (21.6)	46 (18.5)	
Index of Multiple Deprivation (units)	7.7 (6.4, 11.6)	7.7 (6.4, 11.7)	0.63
Completion of questionnaire (post-menstrual weeks)	40.7 ± 9.4	41.4 ± 10.3	0.56
**Female infants**	**n = 148**	**n = 253**	
Gestation (weeks)	40.1 ± 1.2	39.7 ± 1.6	**0.004***
Birth weight (kg)	3.45 ± 0.44	3.36 ± 0.56	0.07
Birth AGD (cm)[c]	0.90 ± 0.26	0.91 ± 0.29	0.59

Values are mean ± SD or median (interquartile range) for continuous variables, *n* (%) for categorical variables.

[a]*P* values: comparing exposed and unexposed mothers/infants. Mann–Whitney *U* test for pre-pregnancy BMI and Index of Multiple Deprivation; t-test for other continuous variables; chi-square test for categorical variables (with Yates' continuity correction for 2 × 2 tables).

[b]Data on ethnicity are missing for 39 mothers.

[c]Data on birth AGD are missing for 124 infants.

*$P < 0.05$ for exposed versus not exposed.
